# Biplanar Versus Triplanar Chevron Osteotomy for the Correction of Hallux Valgus: A Comparison of Radiologic Outcomes

**DOI:** 10.7759/cureus.107753

**Published:** 2026-04-26

**Authors:** Kaylem M Feeney, Lester D'Souza

**Affiliations:** 1 Orthopedics, University of Limerick School of Medicine, Galway, IRL; 2 Orthopedics, University Hospital Limerick, Limerick, IRL

**Keywords:** chevron osteotomy, deformity, hallux valgus, orthopaedics, osteotomy, podiatry

## Abstract

Introduction

Hallux valgus is a common disorder of the foot. The chevron osteotomy is among the most common methods of surgically correcting mild to moderate hallux valgus, though it has been associated with inadequate distal metatarsal articular angle (DMAA) correction and a risk of hallux valgus recurrence. This study aimed to compare the effectiveness of the triplanar and biplanar chevron osteotomies in correcting mild to moderate hallux valgus. Specifically, we aimed to determine if the triplanar chevron osteotomy results in superior correction of the DMAA compared to the biplanar chevron osteotomy.

Methods

A retrospective review of patient medical charts and preoperative and postoperative radiographs was performed. A total of 55 patients were included, with 28 patients in the biplanar chevron group and 27 patients in the triplanar chevron group. The DMAA and intermetatarsal (IM) angles were measured on preoperative and postoperative radiographs. Statistical analysis was carried out on SPSS.

Results

The DMAA and IM angles improved significantly in both groups (p = <0.001). There was no significant difference in the mean postoperative IM angle in the biplanar versus triplanar groups (9.58 degrees versus 9.19 degrees, respectively, p = 0.279). However, there was a significant difference in the mean postoperative DMMA in the triplanar versus biplanar groups (7.88 degrees versus 8.79 degrees, respectively, p = 0.026).

Conclusions

The biplanar and triplanar chevron osteotomies are equally effective in reducing IM angle in mild to moderate hallux valgus. The triplanar chevron osteotomy significantly increases DMAA correction when compared to the biplanar chevron osteotomy and may therefore reduce hallux valgus recurrence.

## Introduction

Hallux valgus is a common disorder of the foot, affecting approximately 35% of those over the age of 65 [1,2]. While conservative treatment may temporarily relieve pain or discomfort related to hallux valgus, definitive treatment is largely surgical. Among the 100 surgical procedures described for the correction of hallux valgus, the chevron osteotomy is among the most frequently used procedures by foot and ankle surgeons worldwide [3]. The chevron osteotomy, first described in 1981 by Austin and Leventin, has long been used in the management of mild to moderate hallux valgus [4]. The traditional chevron osteotomy was composed of a simple “V” horizontal osteotomy and resulted in satisfactory clinical and radiological results [5]. However, some of the problems with the traditional chevron osteotomy included malunion, first ray shortening, and inadequate correction of the distal metatarsal articular angle (DMAA) [3,5]. In order to overcome these issues, the traditional chevron osteotomy was modified to the biplanar chevron osteotomy [6]. The biplanar chevron osteotomy is a modification of the traditional chevron osteotomy. The difference between the traditional chevron osteotomy and the biplanar chevron osteotomy is an alteration of the angle of the saw when carrying out the cut of the plantar limb. In the biplanar chevron osteotomy, the plantar limb is cut at 90 degrees to the vertical cut with the saw held at an angle, in a dorso-medial to plantar-lateral direction, similar to when performing a scarf osteotomy [7]. The aim of this modification was to account for the inherent 1mm shortening of the first metatarsal during a chevron osteotomy. This modification aimed to restore the full weight-bearing function of the first metatarsal head and therefore prevent transfer metatarsalgia [8].

In follow-up studies, the biplanar chevron osteotomy proved to be at least as effective in terms of clinical outcomes and its ability to radiologically correct hallux valgus deformity [6]. In addition, the biplanar chevron osteotomy had the additional benefit of accounting for first ray shortening and subsequently reducing the risk of transfer metatarsalgia compared to the traditional chevron osteotomy [6]. However, much like the traditional chevron osteotomy, the biplanar chevron osteotomy was still associated with inadequate correction of the DMAA and subsequent recurrence of hallux valgus deformity.

The estimated recurrence rate of hallux valgus following surgery is 24.86%, and an inadequate correction of the DMAA has been suggested as a potential risk factor for hallux valgus recurrence postoperatively [9-12].

The triplanar chevron osteotomy was described in 2007 [7]. The triplanar chevron osteotomy is a modification of the biplanar chevron osteotomy. This modification introduced a medial closing wedge in addition to the “V” osteotomy, and lateral and plantar translation of the fragments. The proposed benefit of the triplanar chevron osteotomy over the biplanar chevron osteotomy was that the addition of a medial closing wedge osteotomy would result in superior correction of the DMAA and therefore reduce the risk of hallux valgus recurrence postoperatively.

While this has been hypothesized, no study to date has compared the effectiveness of biplanar versus triplanar chevron osteotomy in reducing the DMAA. Therefore, the aim of this retrospective comparative study was to compare the radiological outcomes of the biplanar osteotomy versus the triplanar chevron osteotomy in the management of mild to moderate hallux valgus. Specifically, the aim of this study was to review and compare the radiological outcomes of DMAA and intermetatarsal (IM) angle postoperatively between patients undergoing biplanar and triplanar chevron osteotomies for hallux valgus correction. We hypothesized that the addition of the medial wedge closing osteotomy in the triplanar chevron osteotomy would result in a superior correction of the DMAA when compared with a biplanar chevron osteotomy.

This article was previously posted to the Research Square preprint server on June 13, 2023 [13].

## Materials and methods

This study was a retrospective analysis of medical charts and radiographs of patients who underwent either a biplanar (group 1) or a triplanar (group 2) chevron osteotomy for the correction of hallux valgus. Institutional review board approval was obtained (registration number 100/2022) prior to the initiation of this study.

Patient selection

All patients over the age of 18 who underwent either a biplanar or triplanar chevron osteotomy for hallux valgus correction under the care of the senior author were eligible for inclusion. Exclusion criteria included patients who had failed previous surgery for hallux valgus of the same foot and patients with inflammatory arthritis. Patients were identified by reviewing the theater lists of the senior author between 2017 and 2018. Radiographs and medical charts of patients who underwent either a biplanar or triplanar chevron osteotomy for hallux valgus correction were retrieved and reviewed by the first author. All procedures were performed as described above by the senior author of this study. As the DMAA is considered to be non-pathological up to 10 degrees, those with a smaller DMAA underwent biplanar chevron osteotomy, while those with a large DMAA underwent triplanar chevron osteotomy [2,14,15].

Data collection

Demographics

Demographic data including gender, age, and which foot was operated on were gathered from each patient’s medical charts.

Radiologic Measurements

Preoperative and postoperative DMAA and IM angles were calculated on both preoperative and postoperative radiographs by the first author. Measurements were repeated on two separate occasions and confirmed by the senior author to improve accuracy.

**Figure 1:**
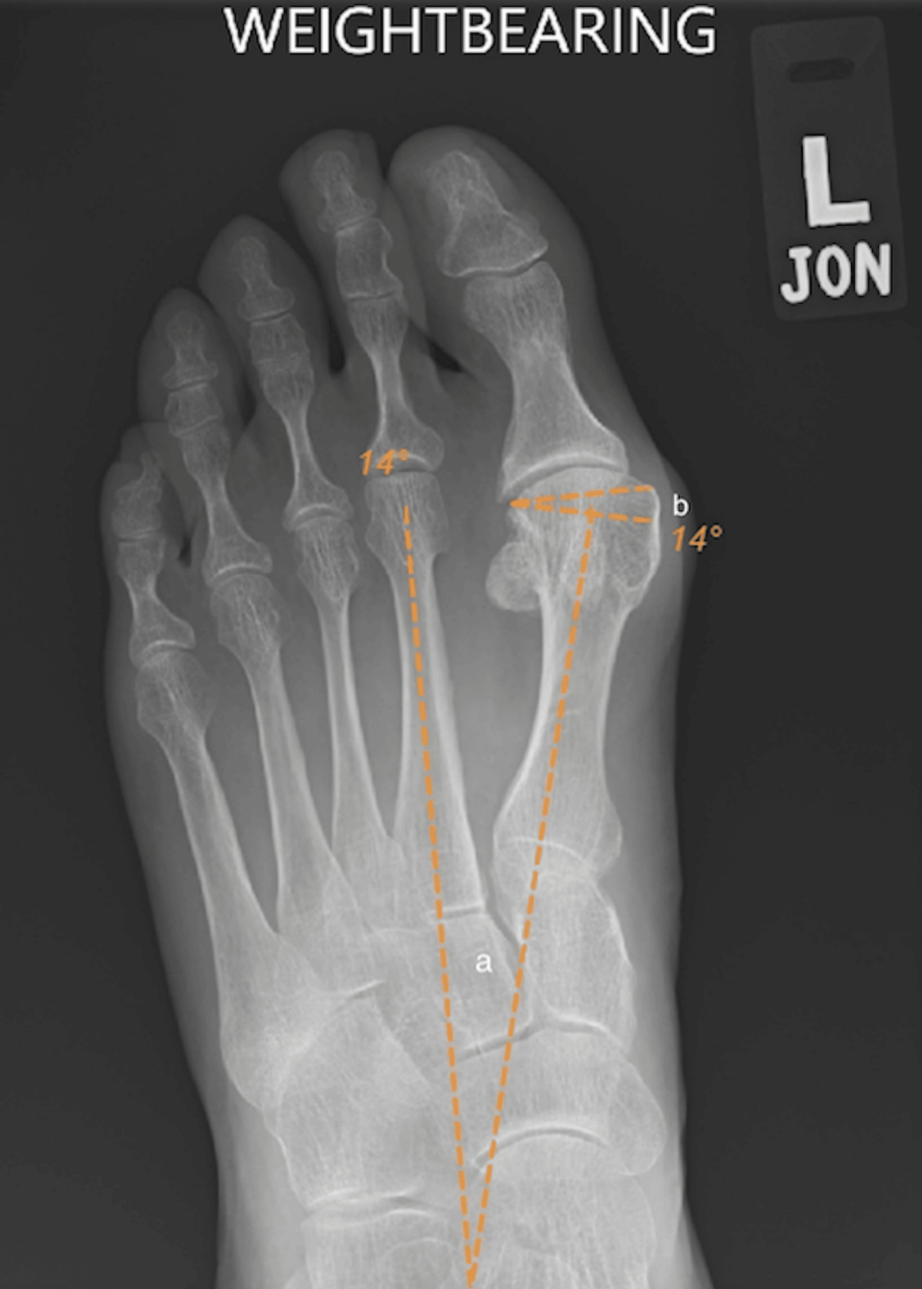
Preoperative weight-bearing anteroposterior X-ray of the left foot The figure demonstrates a preoperative IM angle (a) of 14 degrees and DMAA (b) of 14 degrees prior to triplanar chevron osteotomy. DMAA, distal metatarsal articular angle; IM, intermetatarsal

**Figure 2:**
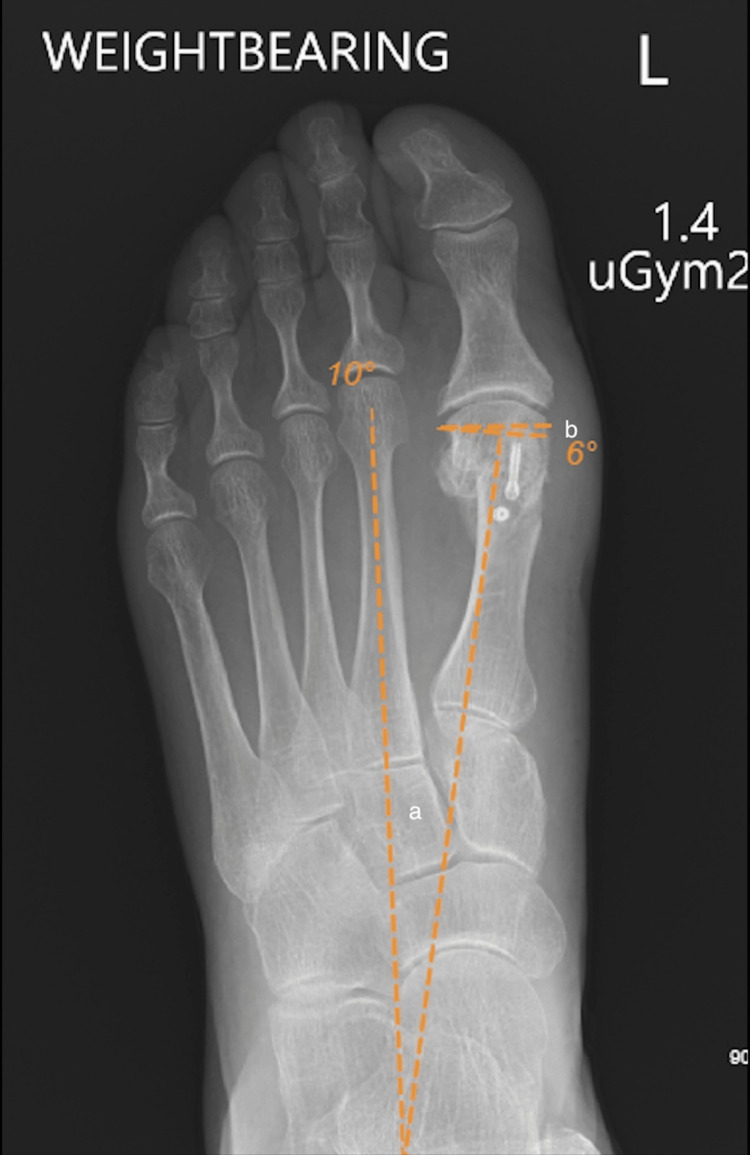
Postoperative weight-bearing anteroposterior X-ray of the left foot The figure demonstrates the reduced IM angle (a) of 10 degrees and DMAA (b) of 6 degrees following triplanar chevron osteotomy. DMAA, distal metatarsal articular angle; IM, intermetatarsal

The DMAA is defined as the angle formed by a line perpendicular to the long axis of the first metatarsal and a line drawn parallel to the distal articular surface of the first metatarsal [2,13,14]. The DMAA is considered non-pathological if it is less than 10 degrees [2]. The IM angle was measured as the angle formed by the bisection of the first metatarsal and the diaphyseal portions of the second metatarsal [2,15]. A normal IM angle is 9 degrees or less [2,15,16]. An example of radiological measurements showing the DMAA and IM angles from preoperatively to postoperatively can be seen in Figures 1, 2.

Statistical analysis

Sample size calculation was performed using the G*Power software. A two-tailed independent samples t-test a priori power analysis was performed with the alpha set at 0.05 and the power set at 0.8. With an effect size of 0.8, the required sample size was calculated to be 52 participants (26 in each group). Statistical analysis was conducted using SPSS Version 26 (IBM Corp., Armonk, NY). Descriptive statistics were performed to describe demographic details. The paired t-test was used to compare the mean difference in angles within each group preoperatively and postoperatively. The independent samples t-test was used to compare the mean difference in demographic data and measured angles between the two groups preoperatively and postoperatively. The statistical significance level was set to a p-value of <0.05 with a 95% confidence interval (CI).

## Results

A total of 55 feet in 55 patients met criteria for inclusion. As can be seen in Table 1, 27 (96.4%) of patients in the biplanar group were female, while one (3.6%) patient was male. In the triplanar group, 25 (92.6%) were female, while two (7.4%) were male. There was no significant difference in the mean age of participants in the biplanar chevron group compared to the triplanar group (56.9 versus 60.9 years, respectively, p = 0.356). In the biplanar group, surgery was performed on the left foot in 12 (42.9%) cases, while surgery was performed on the right foot in 16 (57.1%) cases. In the triplanar group, surgery was performed on the right foot in 12 (44.4%) cases, while surgery was performed on the left foot in 15 (55.6%) cases. No significant difference was observed between groups for any demographic details. Full demographic details, broken down by group, are displayed in Table 1.

**Table 1:** Demographic data of participants Descriptive statistics were used to describe demographic details. The p-value is reported from the paired t-test. *Significance level was set to a p-value of 0.05. n.s., non-significant

	Biplanar chevron (n = 28)	Triplanar chevron (n = 27)	p-Value for overall difference*
Male, n (%)	1 (3.6)	2 (7.4)	n.s.
Female, n (%)	27 (96.4)	25 (92.6)	n.s.
Age, mean±SD	56.9±16.6	60.9±15.1	n.s.
Left foot, n (%)	12 (42.9)	12 (44.4)	n.s.
Right foot, n (%)	16 (57.1)	15 (55.6)	n.s.

Pre- and postoperative radiological outcomes are presented in Tables 2, 3. There was no significant difference in the mean preoperative IM angle in the biplanar versus triplanar groups (14.04 degrees versus 14.19 degrees, respectively, p = 0.521). There was a significant difference in preoperative DMAA in the triplanar chevron versus the biplanar chevron groups (16.37 degrees versus 11.04 degrees, respectively, p = <0.001). There was no significant difference in the mean postoperative IM angle in the biplanar versus triplanar groups (9.58 degrees versus 9.19 degrees, respectively, p = 0.279). There was a significant difference in the mean postoperative DMMA in the triplanar versus biplanar groups (7.88 degrees versus 8.79 degrees, respectively, p = 0.026) (Table 2).

**Table 2:** Between-group analysis of pre- and postoperative radiological data The p-value is reported from the independent samples t-test. *Significance level was set to a p-value of 0.05. **Statistically significant. DMAA, distal metatarsal articular angle; IM, intermetatarsal

	Biplanar chevron	Triplanar chevron	p-Value for overall difference*
Preoperative			
IMA, mean±SD	14.04±0.84	14.19±0.87	0.521
DMAA, mean±SD	11.04+1.26	16.37±1.97	<0.001**
Postoperative			
IMA, mean±SD	9.58±1.2	9.19±1.42	0.279
DMAA, mean±SD	8.79±1.17	7.88±1.69	0.026**

**Table 3:** Within-group analysis of pre- and postoperative radiological data 95% CIs and p-values are reported from the paired samples t-test. All angles are listed in degrees. *Significance level was set to a p-value of 0.05. **Statistically significant. DMAA, distal metatarsal articular angle; IM, intermetatarsal

	Preoperative	Postoperative	Mean difference	SD	CI	p-Value for overall difference*
Biplanar chevron						
IM angle, mean±SD	14.04±0.84	9.58±1.2	4.46	1.35	3.94 – 4.99	<0.001**
DMAA, mean±SD	11.04±1.26	8.79±1.17	2.25	1.08	1.83 – 2.67	<0.001**
Triplanar chevron						
IM angle, mean±SD	14.19±0.87	9.19±1.42	5.0	1.59	4.37 – 5.63	<0.001**
DMAA, mean±SD	16.37±1.97	7.88±1.69	8.49	2.24	7.59 – 9.37	<0.001**

There was a significant improvement in IM angle from preoperatively to postoperatively in both the biplanar chevron group (14.04 to 9.58 degrees; mean difference 4.46±1.35 degrees; 95% CI 3.94 - 4.99; p = <0.001) and the triplanar chevron group (14.19 to 9.19 degrees; mean difference 5.0±1.59 degrees; 95% CI 4.37 - 5.63; p = <0.001). In addition, there was a significant improvement in DMMA from preoperatively to postoperatively in both the biplanar chevron group (11.04 to 8.79 degrees; mean difference 2.25±1.08 degrees; 95% CI 1.83 - 2.67; p = <0.001) and the triplanar chevron group (16.37 to 7.88 degrees, mean difference 8.49±2.24 degrees, p = <0.001) (Table 3).

## Discussion

This was a retrospective study to compare the effectiveness of the biplanar chevron osteotomy versus the triplanar chevron osteotomy in the management of mild to moderate hallux valgus. Specifically, we aimed to determine if a triplanar chevron osteotomy resulted in superior correction of the DMAA when compared to the biplanar chevron osteotomy.

Since the chevron osteotomy was first described in 1981, many studies have evaluated its effectiveness in the management of hallux valgus [4,17,18]. It has typically been used for the management of mild to moderate hallux valgus in patients with an IM angle of 15 degrees or less without osteoarthritis of the first metatarsophalangeal joint. For patients with an IM angle of greater than 15 degrees, a more proximal osteotomy such as the scarf osteotomy is more frequently performed [2]. In high-quality studies including randomized controlled trials and systematic reviews, the chevron osteotomy has proven to be a safe and effective procedure for managing mild to moderate hallux valgus [19]. Studies have demonstrated that the chevron osteotomy successfully and predictably reduces the IM and hallux valgus angles in mild to moderate hallux valgus [18-22]. In addition, the chevron osteotomy results in a significant improvement in patient-reported outcome measures such as the VAS (visual analog scale) and AOFAS (American Orthopaedic Foot & Ankle Society) scores at both short and long-term follow-up [18-22].

Our study has demonstrated that a biplanar chevron osteotomy was associated with a mean reduction in IM angle of 4.46 degrees (95% CI 3.94 to 4.99, p = <0.001), which was statistically significant. Similarly, a triplanar chevron osteotomy was associated with a mean reduction in IM angle of 5 degrees (95% CI 4.37 to 5.63, p = <0.001), which was statistically significant. There was no significant difference in postoperative IM angle between each group postoperatively (p = 0.279). These results are consistent with the findings in the current literature. A systematic review of 25 studies including a total of 1,029 patients published in 2012 showed that the chevron osteotomy significantly reduces the IM angle by a mean of 5.33 degrees (95% CI, 5.12 to 5.54, p = <0.001) [19]. A more recent large cohort study by van Groningen et al. demonstrated a significant mean reduction in IM angle of 6.1 degrees (95% CI 5.9 to 6.4, p = <0.001) following chevron osteotomy for hallux valgus correction [23]. Our findings are therefore consistent with the current literature and suggest that both a biplanar and triplanar modification of the chevron osteotomy are equally effective in reducing IM angle toward normal in mild to moderate hallux valgus deformity.

As demonstrated in Figures 1, 2, the DMAA is the angle formed by drawing a perpendicular line to the long axis of the first metatarsal and the distal articular surface of the first metatarsal [2,14,15]. In hallux valgus, the DMAA describes the valgus angulation of the distal articular surface of the first metatarsal head. The DMAA is important in assessing hallux valgus deformity because valgus angulation of the distal articular surface of the first metatarsal head is one of the most frequently cited bone deformities in hallux valgus [24-25]. While the existence of the DMAA and the role of the DMAA in hallux valgus development and progression have been questioned by some authors in recent years, a recent high-quality comparative study using 3D weight-bearing CT has confirmed that valgus deformity of the articular surface of the first metatarsal head is present in those with hallux valgus when compared to control subjects [25].

In a recent systematic review, the prevalence of hallux valgus recurrence following surgery ranged from 9% to 73% across 23 studies including 2,914 individuals, with a pooled prevalence of hallux valgus recurrence of 24.86% following surgery. The variation in reported recurrence rates most likely represents the substantial variation in the duration of follow-up, definition of what constitutes hallux valgus recurrence, incomplete reporting, surgical technique, and preoperative deformity across studies [26].

Several studies have demonstrated that an increased preoperative DMAA and an inadequately addressed postoperative DMAA, which may occur following inadequate correction of the DMAA during surgery, is associated with a more severe progression of hallux valgus preoperatively and an increased risk of hallux valgus recurrence postoperatively [9-12]. Park and Lee in a study investigating hallux valgus recurrence in patients who had a chevron osteotomy for hallux valgus correction noted that at a mean follow-up of 27.5 months, the immediate postoperative DMAA was much larger in those who had hallux valgus recurrence compared to those who did not have hallux valgus recurrence, suggesting that a larger postoperative DMAA may be associated with a greater risk of hallux valgus recurrence [12].

Our study has demonstrated that the use of a triplanar chevron osteotomy can significantly reduce the postoperative DMAA in mild to moderate hallux valgus. While this study also suggested that the biplanar chevron osteotomy significantly reduces the DMAA in mild to moderate hallux valgus, the mean change was significantly higher in the triplanar group when compared with the biplanar group (Tables 2, 3).

Table 2 also demonstrates that there was a significant difference in the preoperative DMAA between the biplanar and triplanar groups, with the DMAA of the triplanar group being significantly higher than that of the biplanar group. This is because the biplanar chevron osteotomy was used for the correction of hallux valgus in patients with a smaller DMAA, while the triplanar chevron osteotomy was reserved for cases with a larger preoperative DMAA. The fact that the preoperative DMAA was significantly higher in the triplanar chevron group compared to the biplanar chevron group and that the postoperative DMAA was significantly lower in the triplanar chevron group compared to the biplanar chevron group demonstrates that the triplanar chevron osteotomy has the ability to significantly correct the DMAA toward normal. Given that inadequate correction of the DMAA has been associated with hallux valgus recurrence, the authors suggest that the triplanar chevron osteotomy may be more effective than the biplanar chevron osteotomy in reducing the risk of recurrence of hallux valgus in those with a larger preoperative DMAA. However, long-term radiographic follow-up data are required to confirm whether or not achieving adequate DMAA deformity correction truly reduces hallux valgus recurrence in the postoperative period.

The authors suggest that the addition of a medial closing wedge osteotomy in the triplanar chevron osteotomy results in superior correction of large DMAA in mild to moderate hallux valgus and therefore should be considered in the management of hallux valgus with a large DMAA.

Limitations

A major limitation of this research study was the lack of long-term radiographic follow-up. This limitation means that while superior correction of the DMAA can be achieved with a triplanar chevron osteotomy compared to a biplanar chevron osteotomy, it is not possible to confirm whether or not this has reduced recurrence in this cohort. Nevertheless, this study is the first study to demonstrate a significant improvement in DMAA reduction with the addition of a medial wedge closing osteotomy in a triplanar chevron osteotomy when compared with a biplanar chevron osteotomy in the correction of mild to moderate hallux valgus.

## Conclusions

This study analyzed the radiological outcomes following the correction of hallux valgus with either a biplanar or triplanar chevron osteotomy. The results from this study suggest that both the biplanar chevron osteotomy and triplanar chevron osteotomy are equally effective in reducing the IM angle in mild to moderate hallux valgus. In addition, both the biplanar chevron osteotomy and the triplanar chevron osteotomy result in a significant reduction in DMAA. However, our study suggests that the addition of a medial closing wedge osteotomy in the triplanar chevron osteotomy has the potential to increase the correction of the DMAA, particularly in the case of a larger preoperative DMAA, when compared with the biplanar chevron osteotomy. The authors recommend the addition of a medial closing wedge osteotomy to the standard chevron osteotomy in the case of a large DMAA. It is possible that this may reduce the risk of hallux valgus recurrence in the follow-up period. However, further studies with long-term radiographic follow-up are required to determine if superior DMAA correction decreases the likelihood of hallux valgus recurrence postoperatively.

## References

[1] Feeney KM, Kearns SR (2021). A technique to correct troughing in a Z-osteotomy for hallux valgus correction. J Foot Ankle Surg.

[2] Nix S, Smith M, Vicenzino B (2010). Prevalence of hallux valgus in the general population: a systematic review and meta-analysis. J Foot Ankle Res.

[3] Fraissler L, Konrads C, Hoberg M, Rudert M, Walcher M (2016). Treatment of hallux valgus deformity. EFORT Open Rev.

[4] Austin DW, Leventen EO (1981). A new osteotomy for hallux valgus: a horizontally directed "V" displacement osteotomy of the metatarsal head for hallux valgus and primus varus. Clin Orthop Relat Res.

[5] Schneider W, Aigner N, Pinggera O, Knahr K (2004). Chevron osteotomy in hallux valgus. Ten-year results of 112 cases. J Bone Joint Surg Br.

[6] Corte-Real NM, Moreira RM (2009). Modified biplanar chevron osteotomy. Foot Ankle Int.

[7] Ahn J, Lee HS, Seo JH, Kim JY (2016). Second metatarsal transfer lesions due to first metatarsal shortening after distal chevron metatarsal osteotomy for hallux valgus. Foot Ankle Int.

[8] Pearce CJ, Sexton SA, Sakellariou A (2008). The triplanar chevron osteotomy. Foot Ankle Surg.

[9] Pentikainen I, Ojala R, Ohtonen P, Piippo J, Leppilahti J (2014). Preoperative radiological factors correlated to long-term recurrence of hallux valgus following distal chevron osteotomy. Foot Ankle Int.

[10] Okuda R, Kinoshita M, Yasuda T, Jotoku T, Kitano N, Shima H (2007). The shape of the lateral edge of the first metatarsal head as a risk factor for recurrence of hallux valgus. J Bone Joint Surg Am.

[11] Lee SY, Chung CY, Park MS (2018). Radiographic measurements associated with the natural progression of the hallux valgus during at least 2 years of follow-up. Foot Ankle Int.

[12] Park CH, Lee WC (2017). Recurrence of hallux valgus can be predicted from immediate postoperative non-weight-bearing radiographs. J Bone Joint Surg Am.

[13] Feeney KM, D'Souza LG (2023). Biplanar versus triplanar chevron osteotomy for the correction of hallux valgus: a comparison of radiologic outcomes [PREPRINT]. Research Square.

[14] Richardson EG, Graves SC, McClure JT, Boone RT (1993). First metatarsal head-shaft angle: a method of determination. Foot Ankle.

[15] Coughlin MJ, Freund E (2001). The reliability of angular measurements in hallux valgus deformities. Foot Ankle Int.

[16] Smith RW, Reynolds JC, Stewart MJ (1984). Hallux valgus assessment: report of research committee of American Orthopaedic Foot and Ankle Society. Foot Ankle.

[17] Piqué-Vidal C, Vila J (2009). A geometric analysis of hallux valgus: correlation with clinical assessment of severity. J Foot Ankle Res.

[18] Deenik AR, Pilot P, Brandt SE, van Mameren H, Geesink RG, Draijer WF (2007). Scarf versus chevron osteotomy in hallux valgus: a randomized controlled trial in 96 patients. Foot Ankle Int.

[19] Clemente P, Mariscal G, Barrios C (2022). Distal chevron osteotomy versus different operative procedures for hallux valgus correction: a meta-analysis. J Orthop Surg Res.

[20] Smith SE, Landorf KB, Butterworth PA, Menz HB (2012). Scarf versus chevron osteotomy for the correction of 1-2 intermetatarsal angle in hallux valgus: a systematic review and meta-analysis. J Foot Ankle Surg.

[21] Fukushi JI, Tanaka H, Nishiyama T (2022). Comparison of outcomes of different osteotomy sites for hallux valgus: A systematic review and meta-analysis. J Orthop Surg (Hong Kong).

[22] Klugarova J, Hood V, Bath-Hextall F, Klugar M, Mareckova J, Kelnarova Z (2017). Effectiveness of surgery for adults with hallux valgus deformity: a systematic review. JBI Database System Rev Implement Rep.

[23] van Groningen B, van der Steen MC, Reijman M, Bos J, Hendriks JG (2016). Outcomes in chevron osteotomy for hallux valgus in a large cohort. Foot (Edinb).

[24] Coughlin MJ (1995). Juvenile hallux valgus: etiology and treatment. Foot Ankle Int.

[25] Lalevée M, Barbachan Mansur NS, Lee HY (2022). Distal metatarsal articular angle in hallux valgus deformity. Fact or fiction? A 3-dimensional weightbearing CT assessment. Foot Ankle Int.

[26] Ezzatvar Y, López-Bueno L, Fuentes-Aparicio L, Dueñas L (2021). Prevalence and predisposing factors for recurrence after hallux valgus surgery: a systematic review and meta-analysis. J Clin Med.

